# Novel Translational Concept: Axon-to-Muscle Exosomal Signaling as an Emerging Therapeutic Target in Spinal Muscular Atrophy

**DOI:** 10.3390/biomedicines13122876

**Published:** 2025-11-25

**Authors:** Almir Fajkić, Andrej Belančić, Yun Wah Lam, Valentino Rački, Kristina Pilipović, Tamara Janković, Silvestar Mežnarić, Jasenka Mršić-Pelčić, Dinko Vitezić

**Affiliations:** 1 Department of Pathophysiology, Faculty of Medicine, University of Sarajevo, 71000 Sarajevo, Bosnia and Herzegovina; 2 Department of Basic and Clinical Pharmacology with Toxicology, Faculty of Medicine, University of Rijeka, Brace Branchetta 20, 51000 Rijeka, Croatia; kristina.pilipovic@medri.uniri.hr (K.P.); tamara.jankovic@medri.uniri.hr (T.J.); silvestar.meznaric@medri.uniri.hr (S.M.); jasenka.mrsic.pelcic@medri.uniri.hr (J.M.-P.); dinko.vitezic@medri.uniri.hr (D.V.); 3 Department of Health Sciences, School of Nursing and Health Sciences, Hong Kong Metropolitan University, Hong Kong SAR, China; wywlam@hkmu.edu.hk; 4 Department of Neurology, Clinical Hospital Center Rijeka, Kresimirova 42, 51000 Rijeka, Croatia; valentino.racki@medri.uniri.hr

**Keywords:** spinal muscular atrophy, exosomes, axon-to-muscle signaling, neuromuscular junction, translational therapy

## Abstract

Spinal muscular atrophy (SMA) has transitioned from a uniformly fatal disease to a treatable condition, yet incomplete neuromuscular recovery underscores the limits of current SMN-restorative therapies. Emerging data implicate disrupted axon-to-muscle exosomal signaling as an important, overlooked driver of residual dysfunction. Exosomes, nanovesicles mediating bidirectional neuronal-muscular communication, carry synaptic organizers, trophic factors, and microRNAs essential for neuromuscular junction integrity. SMN deficiency alters exosomal biogenesis and cargo, leading to loss of agrin-MuSK signaling, impaired β-actin transport, and muscle atrophy. Comparative insights from amyotrophic lateral sclerosis and muscular dystrophy reveal that stem-cell-derived or engineered exosomes restore synaptic stability, enhance regeneration, and cross biological barriers safely. Thus, we speculate herein on a translational model integrating exosome-based therapies with existing genetic interventions to achieve durable, systems-level recovery in SMA. Exosomal profiling may further yield minimally invasive biomarkers for disease monitoring and treatment optimization, establishing vesicle-mediated communication as a novel therapeutic axis in neuromuscular medicine.

## 1. Introduction

Spinal muscular atrophy (SMA) is a rare, inherited neuromuscular disorder with an incidence of approximately 1 in 10,000 live births and a prevalence of 1–2 per 100,000 individuals. SMA imposes a substantial clinical and societal burden despite its classification as a rare disease [1,2]. The most common form, 5q-linked SMA, results from homozygous deletions or mutations in the survival motor neuron 1 (SMN1) gene on chromosome 5q13. A nearly identical paralogue, SMN2, partially compensates for SMN1 loss by producing limited amounts of functional SMN protein. However, a single nucleotide substitution in SMN2 disrupts splicing, causing most transcripts to exclude exon 7 and produce a truncated, unstable protein, thereby generating a profound deficiency of full-length SMN [1].

The SMN protein is indispensable for the assembly of small nuclear ribonucleoproteins (snRNPs) and pre-mRNA splicing, but its cellular functions extend far beyond RNA metabolism [1,3,4]. SMN is critical for axonal mRNA trafficking, cytoskeletal organization, mitochondrial homeostasis, and proteostasis, all essential for motor neuron survival [5,6,7,8]. Although ubiquitously expressed, SMN deficiency disproportionately affects lower motor neurons in the spinal cord, resulting in progressive, symmetrical muscle weakness, atrophy, and eventual loss of motor function [1,9].

Clinically, SMA manifests along a continuum of severity. At the most severe end, affected neonates exhibit profound hypotonia and early death (type 0 and type 1), whereas later-onset forms allow partial or full ambulation and survival into adulthood (e.g., type 3). Traditionally, these types have been classified based on age at onset, maximum motor function achieved, and typical prognosis (Table 1). This clinical heterogeneity reflects differences in SMN protein levels, largely influenced by SMN2 copy number and additional genetic modifiers [10,11,12].

Over the past decade, the therapeutic landscape of SMA has undergone a paradigm shift. Disease-modifying therapies have transformed SMA from a uniformly fatal infantile disorder into a chronic, manageable condition. Consequently, SMA phenotypes are becoming increasingly variable, and patient outcomes more favorable [13,14]. Nusinersen, an antisense oligonucleotide, promotes exon 7 inclusion in SMN2 transcripts, augmenting SMN protein in the central nervous system. Onasemnogene abeparvovec, a gene therapy, delivers a functional SMN1 gene via adeno-associated virus serotype 9 to motor neurons. Risdiplam, an orally administered small molecule, systemically modifies SMN2 splicing to enhance full-length SMN expression. These therapies have improved survival, motor function, and quality of life, particularly when administered presymptomatically [15,16,17].

Nevertheless, significant limitations remain. SMN restoration alone does not fully prevent or reverse the diverse pathogenic processes triggered by SMN deficiency. Patients on therapy often continue to experience residual motor deficits, neuromuscular junction immaturity, fatigability, and systemic metabolic complications. Pathological hallmarks such as glial dysfunction, mitochondrial impairment, and defective axon-to-muscle signaling persist, underscoring that SMN restoration is necessary but not sufficient for complete functional recovery [18]. Additional challenges include long-term durability, repeated administration for some modalities, and barriers related to cost or healthcare infrastructure [17].

As SMA transitions into a chronically managed condition, attention is shifting toward next-generation strategies that extend beyond SMN replacement [19]. One promising but underexplored area is axon-to-muscle exosomal signaling [20,21,22,23,24]. Exosomes, small extracellular vesicles (EVs) secreted by neurons, muscle cells, and glia, mediate intercellular transfer of proteins, RNAs, and lipids, regulating neuromuscular junction stability, axonal maintenance, and muscle regeneration [25,26,27,28,29]. SMN deficiency may disrupt exosome composition and release, impairing communication along the neuromuscular axis. Therapeutic modulation of exosomal signaling could therefore preserve synaptic integrity, enhance muscle repair, and complement existing SMN-restorative therapies, potentially offering a more holistic approach to disease management.

In this narrative review, we present the translational concept of modulating axon-to-muscle exosomal signaling as a therapeutic target. Given the scoping and exploratory nature of this narrative review, we conducted a comprehensive search of PubMed and Scopus up to the end of September 2025. Due to the specificity and emerging nature of the topic, no strict inclusion or exclusion criteria were applied. Instead, studies were selected based on relevance to exosome biology, axon and muscle signaling, and neuromuscular mechanisms in spinal muscular atrophy. The search used a focused set of key terms, including “spinal muscular atrophy,” “SMA,” “exosome,” “extracellular vesicle (EV),” “neuromuscular junction (NMJ),” “axon”, “muscle signaling,” and “SMN deficiency.” Both preclinical, clinical, and translational evidence were included, with additional articles identified through citation tracking.

To deduce, by complementing existing SMN-restorative therapies, this approach could address downstream pathological features and pave the way for combination strategies capable of delivering more durable and comprehensive clinical benefit to individuals living with SMA.

## 2. Neuromuscular Crosstalk: Beyond Classical Neurotransmission

### 2.1. Conventional NMJ Signaling

The neuromuscular junction (NMJ) is a highly specialized synapse between an α-motor neuron and a skeletal muscle fiber that enables the transmission of an electrical signal from the somatic nervous system to the muscle, ultimately triggering an action potential and subsequent muscle contraction [9,30]. This process is fundamental for voluntary movement and is orchestrated through a complex of structural and molecular processes [30].

Structurally, NMJ is composed of a presynaptic terminal of the motor neuron, the synaptic cleft, and a postsynaptic membrane on the muscle fiber. The presynaptic terminal is characterized by synaptic boutons densely packed with synaptic vesicles filled with the neurotransmitter acetylcholine (ACh). Acetylcholine, synthesized from choline and acetyl-CoA by choline acetyltransferase enzyme, is tightly packed into synaptic vesicles through the action of vesicular acetylcholine transporter (VAChT), and concentrated near the release sites [31]. Upon the arrival of an action potential to the presynaptic terminal, voltage-gated calcium channels open, and Ca^2+^ influx enables fusion of synaptic vesicles with the presynaptic membrane and exocytosis of ACh into the synaptic cleft [32]. The synaptic cleft, the gap between the presynaptic neuron and muscle fiber, is filled with a specialized extracellular matrix known as the synaptic basal lamina, primarily composed of laminins and collagens that contribute to synaptic stability and signaling [33,34]. Notably, basal lamina provides a binding site for acetylcholinesterase (AChE), an enzyme essential for rapid ACh degradation and signal transmission termination [35]. The postsynaptic membrane, also known as the motor endplate, features extensive folds that increase surface area available for neurotransmitter reception [36]. These junctional folds contain densely distributed nicotinic acetylcholine receptors (nAchRs), ligand-gated ion channels, that upon ACh binding allow fast sodium ions influx, leading to depolarization of the muscle membrane and generation of the endplate potential [36]. Voltage-gated Na^+^ channels, concentrated in the junctional folds, further boost excitability [31,37].

In addition to neuronal and muscular components, perisynaptic or terminal Schwann cells envelop the NMJ and are essential not only during NMJ development [38], but also have an important role in the ongoing maintenance of synaptic integrity throughout adulthood [18]. Terminal Schwann cells are active participants in synapse formation, synaptic transmission, plasticity, which adapt the NMJ in physiological and pathological environments [38,39,40].

In essence, NMJ is a fine-tuned synaptic complex that includes neuronal, muscular, and glial components, which ensure precise timing and signal transmission regulation, allowing highly regulated anterograde and retrograde neuromuscular communication [31]. Signaling involves continuous remodeling of both presynaptic motor neurons and postsynaptic muscle fibers, with additional modulation by surrounding glial cells. For example, evidence demonstrates that the regulation of nAChR clustering is mediated not only by motoneurons but also by the innervated muscle fibers. Motoneurons can secrete agrin, which binds to low-density lipoprotein receptor-related protein 4 (Lrp4) in the cell membrane and cytoplasm of myocytes [41], activating muscle-specific kinase (MuSK) which promotes postsynaptic nAChR clustering [42].

### 2.2. Exosomes as Signaling Entities in Neuromuscular Systems

Exosomes are nanoscale membrane vesicles ranging from 30 to 150 nm in diameter [43], originating from the endosomal pathway following the fusion of multivesicular bodies (MVB) with the plasma membrane [44,45]. Structurally, they consist of a lipid bilayer [46] and function as important mediators of cell-to-cell communication under both physiological and pathological conditions [47]. Due to their endogenous origin, exosomes exhibit lower toxicity profiles than synthetic nanoparticles, facilitating efficient delivery of proteins, lipids, and nucleic acids to target cells. Therefore, exosomes have been recognized as powerful vehicles for biomedical applications [48]. Initial studies have associated exosomes as a mechanism for the clearance of cellular debris [49]. However, exosomes are now known to be secreted by nearly all cell types and play diverse roles in regulating cellular responses between the central and peripheral nervous systems [48].

Exosome biogenesis begins with the formation of early endosomes, which subsequently mature into late endosomes or exchange their content with other intracellular organelles. Invagination of late endosomal membranes creates MVBs that either fuse with lysosomes or autophagosomes for degradation or are shuttled along the microtubule system toward the cell surface, where they are secreted through exocytosis as exosomes [43,44]. The formation and release of exosomes are highly regulated processes involving multiple molecular players and mechanisms, both dependent on and independent of the endosomal sorting complex required for transport (ESCRT) complex [43,45]. It is thought that the composition of exosomal cargo can modify its own biogenesis, but such mechanisms remain incompletely understood [43].

Studies have demonstrated that exosomes play a pivotal role in neuromuscular junction (NMJ) formation. Korkut et al. [50] detected that presynaptic motor neurons mediate retrograde signaling through the anterograde delivery of synaptotagmin 4 to postsynaptic cells via exosomes. In addition, exosomes carry important morphogens such as Wnt proteins [51,52,53], bone morphogenic factor (BMP), and regulatory microRNAS (miRNAs), orchestrating the coordinated remodeling of both the presynaptic motor neuron terminal and the postsynaptic muscle fiber membrane [54]. Cargo derived from soluble EVs has been shown to initiate the agrin–Lrp4–MuSK pathway, important for nAChR clustering at the postsynaptic membrane [54].

Wnts, characterized by their high lipophilicity, act as morphogens that regulate NMJ formation via nAChR clustering through both canonical β-catenin-dependent and -independent pathways [55], and are well suited for delivery via exosomes [54]. For example, Wnt1/Wingless (Wg) binds to the exosomal protein Evennes Interrupted (Evi), to be secreted from the periactive zones of a presynaptic neuronal membrane to the postsynaptic cells in *Drosophila* NMJ [53]. MiRNAs, well-known regulators of gene expression within their cells of origin, when packed within EV, can act as an important mechanism of intercellular communication [56]. Notably, Nesler et al. [57] identified specific miRNAs that control activity-dependent synaptic growth at the *Drosophila* NMJ, including modulation of Wg expression.

Moreover, muscle cell-derived exosomes also transport various proteins, lipids, and RNAs, including miRNAs [58], which have been implicated in NMJ modulation [59]. A recent study by Agostini et al. [55] elucidated that differentiated motor neurons produce extracellular EVs packed with miRNAs and Wnts that promote nAChR clustering in myotubes, a key step in NMJ signal transmission.

## 3. Exosomes in Neuromuscular Disorders: Untapped Therapeutic Potential

Emerging evidence across neuromuscular disorders positions axon-to-muscle exosomal signaling as a plausible therapeutic axis for SMA (Table 2). Foundational ALS studies demonstrated this concept: ADSC-derived exosomes reduce oxidative injury and restore mitochondrial function in SOD1-G93A motor neurons via miRNA-dependent survival pathways [60,61,62], while Lee et al. showed that exosomal cargo can diminish SOD1 aggregation and improve respiratory capacity [63]. Morel et al. further demonstrated that neuronal exosomes modulate astrocytic GLT1 through miRNA-regulated mechanisms, attenuating excitotoxicity relevant to SMA vulnerability [64]. Intranasal delivery studies confirmed that exosomes can bypass the blood-brain barrier and produce distributed neuroprotection [65]. Parallel findings from Duchenne muscular dystrophy (DMD) reinforce these mechanistic principles: MSC-derived exosomes stabilize sarcolemmal structure and restore force generation in mdx mice through transfer of membrane-repair and myogenic cargos [66,67,68]. Complementary work highlights engineered exosomes that exert targeted anti-inflammatory effects and promote M2 macrophage polarization in neuroinflammatory contexts [69,70], illustrating their capacity to modify both neural and muscular compartments in conditions with combined pathology. Together, these insights form a coherent mechanistic template directly applicable to SMA, positioning exosomal signaling as a strategy capable of enhancing motor neuron resilience, modulating glial excitability, and supporting muscle integrity beyond the reach of current SMN-focused therapies.

The rationale for exosomal therapy in SMA arises directly from the disease’s defined molecular lesion and its consequences for axonal biology. SMN loss destabilizes axonal mRNA trafficking and cytoskeletal organization [71,72,73], creating a signaling landscape uniquely sensitive to vesicle-based interventions. Foundational work by Rossoll et al. [74] demonstrated that SMN scaffolds the assembly and anterograde transport of transcripts such as β-actin, a determinant of axonal elongation, growth-cone stability, and synaptic maintenance. This reframed SMA pathology as a disorder of impaired axonal logistics, with direct implications for exosome biogenesis and cargo loading.

Building on this, van der Pol et al. [75] showed that SMA patient fibroblasts and mouse models release exosomes with reduced SMN content alongside increased vesicle output. This dual abnormality–quantitative overproduction and qualitative SMN depletion–provides a mechanistic link between the primary genetic defect and vesicle dysfunction, positioning exosomal SMN levels as a biomarker tightly coupled to the disease state.

The developmental timing of SMA introduces a therapeutic window distinct from adult-onset neuromuscular diseases. Udina et al. [76] demonstrated that SMA models exhibit compensatory axon sprouting in response to slow axon degeneration, indicating that the neuromuscular system retains significant plasticity during early disease. This capacity is substantially reduced in adult-onset conditions, underscoring the importance of intervention timing in SMA.

In parallel, the molecular architecture of SMA pathology aligns naturally with exosomal delivery capabilities. Multiple converging defects–impaired RNA processing, protein aggregation, mitochondrial dysfunction, and synaptic instability–can be simultaneously targeted through coordinated exosomal delivery of SMN protein, regulatory miRNAs, metabolic factors, and membrane-stabilizing components [70,77,78,79].

Translational evidence reinforces this therapeutic trajectory. Nash’s pioneering work [80] established that exosomal SMN content reflects disease burden and that exogenously supplied SMN can be restored via vesicle-mediated delivery. René and Parks [81] demonstrated that engineered EVs deliver functional SMN in vitro and in vivo, addressing critical challenges of cargo stability and targeting. Clinically, nusinersen-treated patients exhibit increased full-length SMN transcripts in serum EVs correlating with treatment response, supporting the utility of EV-based biomarkers for real-time monitoring of therapeutic efficacy [82].

Insights from ALS and Duchenne muscular dystrophy indicate that repeated exosome dosing can sustain neuroprotection [62] and that exosomal cargo profiling may complement SMA management by enabling dynamic monitoring of disease activity and treatment response [62,83].

However, several critical challenges remain in translating axon-to-muscle exosomal signaling into clinical practice for SMA. Standardization of exosome isolation and characterization is still unresolved, with substantial variability between laboratories and no consensus on methods suited for developmental-stage diseases such as SMA. This variability also complicates identification of the optimal therapeutic window, which is tightly constrained by early motor neuron vulnerability and evolving neuromuscular architecture [84,85,86,87]. Integrating exosomal therapeutics with current SMN-directed treatments presents both opportunity and complexity. Existing FDA-approved therapies act at transcriptional or translational levels, while exosomal systems could expand tissue distribution, reduce immunogenicity, and prolong biological activity; however, potential interactions between modalities require rigorous evaluation [88,89,90]. Manufacturing and scalability remain major barriers. Pediatric application demands precise dosing, strict safety thresholds, and highly consistent vesicle preparations, none of which are yet achievable at the clinical scale. Current advances, including bioreactor-based culture and genetically modified producer cells, improve yield but still lack standardized protocols necessary for reproducibility and product quality [49,91]. Regulatory pathways specific to therapeutic exosomes remain underdeveloped, further delaying translation [92,93]. Robust biomarkers are another unmet need. Reliable tools to track exosome biodistribution, dosing response, and target engagement will be essential for optimizing treatment regimens and strengthening regulatory approval frameworks [49].

Despite these challenges, converging evidence from ALS, muscular dystrophies, and SMA itself underscores the transformative potential of targeting axon-to-muscle exosomal signaling. These studies recast neuromuscular degeneration not as a failure of isolated cell populations, but as a breakdown in intercellular communication. This conceptual shift supports the development of therapeutic strategies aimed at restoring vesicle-mediated neuronal-muscular signaling and offers a more integrated framework for future SMA interventions [62,91,94].

**Table 2:** Comparative overview of exosome-based mechanisms, therapeutic applications, and translational insights across neuromuscular disorders.

Author(s) and Year	Central Thesis	Key Positive Insights	Key Limitations	Conclusion
**Anakor et al. (2021)** [60]	Exosomes function as both pathogenic and therapeutic mediators in motor neuron diseases.	Cross the blood-brain barrier; carry diverse RNA/protein cargo; enable minimally invasive biomarkers; allow engineering for targeting.	Possible spread of pathogenic proteins; weak intrinsic targeting; uncertain safety; lack of standardization.	High therapeutic and diagnostic promise but requires controlled engineering and safety validation.
**Bonafede et al. (2016)** [61]	Stromal cell-derived exosomes exert neuroprotection in ALS cell models.	Reduce oxidative stress; support neuronal survival; improve mitochondrial homeostasis.	In vitro only; unclear in vivo mechanisms; no human data.	Promising early evidence; needs in vivo and clinical translation.
**Bonafede et al. (2020)** [62]	ASC-exosomes slow ALS progression in mouse models.	Improve motor function and survival; feasible non-cellular therapy; repeated dosing effective.	Dosing and delivery not optimized; long-term safety unknown; production challenges.	Strong preclinical support for clinical development.
**Leng et al. (2021)** [68]	Exosomes enhance muscle membrane stability in DMD.	Restore muscle integrity; delay progression; multiple feasible cell sources.	No human studies; dosing unresolved; possible off-target effects.	Novel therapeutic avenue for DMD; needs further validation.
**Wu et al. (2025)** [69]	Intranasal MSC-exosomes reduce neuroinflammation and improve outcomes in EAE.	Non-invasive delivery; strong anti-inflammatory effects; broad CNS applicability.	Only animal models; human immune effects unclear; dosing uncertain.	Intranasal route is promising for neuromodulation strategies.
**René & Parks (2025)** [81]	EVs deliver SMN protein across tissue barriers to rescue SMA phenotypes.	Direct protein replacement; bypasses limitations of gene therapy; robust functional rescue in models.	Human safety not tested; production scalability unresolved.	Potential paradigm shift in SMA therapy pending translational steps.
**Trifunov et al. (2023)** [82]	EV-based SMN transcript quantification tracks SMA therapy response.	Enables non-invasive monitoring; supports dose adjustment; may predict outcomes.	Not therapeutic; requires validation; standardization of workflows needed.	Valuable biomarker tool for SMA therapy optimization.
**Chen et al. (2025)** [83]	Muscle-derived EVs mitigate ALS-related muscle inflammation and atrophy.	Anti-inflammatory effects; promote M2 macrophages; suppress NF-κB signaling.	Preclinical only; variability in EV source; translational hurdles.	Multi-mechanistic approach that merits clinical exploration.
**India SCC (2025)** [84]	Early clinical data show functional improvement with exosome therapy in SMA.	Suggests patient-level efficacy; minimally invasive; supports human feasibility.	Preliminary non-RCT data; small samples; potential bias.	Promising clinical signal; high-quality trials required.

ALS—amyotrophic lateral sclerosis; ASC—adipose-derived stromal cell; DMD—Duchenne muscular dystrophy; EAE—experimental autoimmune encephalomyelitis; EV—extracellular vesicle; MSC—mesenchymal stem cell; SMN—survival motor neuron; SMA—spinal muscular atrophy; CNS—central nervous system; NF-κB—nuclear factor kappa-light-chain-enhancer of activated B cells.

## 4. Proposed Hypothesis: Dysfunctional Axon-to-Muscle Exosomal Signaling in SMA

**Figure 1:**
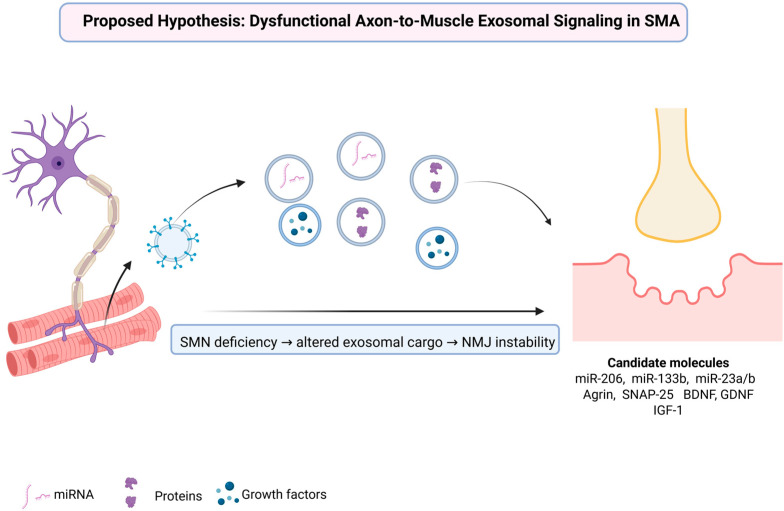
Mechanistic model of altered axon-to-muscle exosomal communication and candidate molecular mediators in spinal muscular atrophy. Created in BioRender. Fajkić, A. (2025) https://BioRender.com/ye2j4l8.

Emerging evidence indicates that SMN deficiency critically disrupts axon-to-muscle exosomal signaling, causing a loss of essential synaptic organizers, trophic factors, and regulatory microRNAs required for NMJ stability and muscle integrity [94,95,96]. Rather than a mere reduction in vesicle release, the pathological hallmark appears to involve altered cargo composition: attenuated delivery of synaptic organizers such as Agrin, reduced levels of vesicular proteins including synaptotagmin-2 and SV2B, and impaired transfer of regulatory microRNAs essential for synaptogenesis and muscle regeneration [54,97]. Concomitantly, maladaptive signals may emerge within EVs, accelerating postsynaptic destabilization and compounding NMJ vulnerability [54]. Together, these findings support a framework in which SMA is reframed not only as a disorder of motor neuron survival but as a disease of failed intercellular communication, where disease progression reflects the collapse of molecular dialogue between motor neurons and muscle fibers [98]. This proposed framework is illustrated in Figure 1.

SMN deficiency in motor neurons disrupts the biogenesis and cargo composition of exosomes released along axons, leading to impaired molecular communication with muscle fibers. The altered exosomal cargo is characterized by a reduced transfer of regulatory miRNAs (miR-206, miR-133b, miR-23a/b), synaptic and vesicular proteins (Agrin, SNAP-25), and growth factors (BDNF, GDNF, IGF-1), all essential for NMJ maturation and maintenance. Loss of these protective signals, together with the emergence of maladaptive exosomal content, contributes to NMJ instability and progressive muscle atrophy in spinal muscular atrophy.

### 4.1. SMN as a Molecular Orchestrator of Vesicle Biology

The SMN protein is canonically recognized for its indispensable role in assembling spliceosomal small nuclear ribonucleoproteins (snRNPs), fundamental to pre-mRNA splicing across all eukaryotic cells [99]. Accumulating evidence now extends SMN’s functional repertoire far beyond RNA splicing, positioning it as a central coordinator of cytoskeletal remodeling, local translation, and vesicular trafficking. At the cytoskeletal interface, SMN accumulates at membrane protrusions during actin filament remodeling and interacts with caveolin-1, a scaffold protein anchoring translation machinery components to the plasma membrane [100]. SMN deficiency disrupts these processes, impairing membrane remodeling, depleting ribosomes from the plasma membrane, and attenuating local translation—underscoring its critical role in cytoskeletal regulation [100].

In parallel, SMN associates with mRNA-binding proteins to assemble and traffic messenger ribonucleoprotein (mRNP) complexes along cytoskeletal tracks, ensuring precise mRNA localization and local translation in axons and dendrites [101]. Defects in this pathway directly contribute to SMA pathogenesis, where impaired RNA transport and local translation compromise early neuronal function even before motor neuron death occurs [102].

Finally, SMN regulates vesicular trafficking and endocytosis. Reduced SMN levels impair endosomal transport, synaptic vesicle docking, and endocytosis in animal models and human cells, leading to synaptic dysfunction that is independent of motor neuron loss [103]. These processes—actin cytoskeleton dynamics, RNA transport, vesicular trafficking—converge in exosome biogenesis, as exosome formation within MVBs requires coordinated membrane remodeling, cytoskeletal scaffolding, and vesicular transport machinery [81]. Thus, SMN sits at the center of a network regulating vesicle production, cargo loading, and release.

### 4.2. Mechanistic Basis: Three Converging Pathways

This disruption manifests through three experimentally supported mechanisms. First, SMN deficiency deprives motor neurons and muscle fibers of essential trophic and synaptic molecules. SMN loss dysregulates synaptogenesis genes—such as the skipping of Agrin Z exons critical for NMJ maintenance—while simultaneously upregulating synapse-pruning factors, including complement C1q, and downregulating transcription factors like Etv1/ER81, which are indispensable for sensory–motor circuitry integrity [104].

Second, SMN loss impairs packaging of motoneuron-specific microRNAs, particularly miR-218, a master regulator of neuromuscular stability whose depletion precipitates NMJ defects and progressive motor neuron loss, phenocopying SMA and ALS [105]. Normally, these microRNAs suppress gene networks that drive neuromuscular failure when derepressed. Defective axonal transport of β-actin mRNA further compromises local translation, amplifying synaptic vulnerability [105].

Third, SMN deficiency misroutes stress-induced RNAs and proteins into exosomes, converting them from neuroprotective carriers into pathogenic vectors that propagate degeneration across the neuromuscular axis. Analogous processes in ALS demonstrate how EVs propagate degeneration across synapses in a non-cell-autonomous manner, implicating altered vesicular content as a shared mechanism of motor neuron disease [106].

Collectively, these findings identify vesicular signaling defects as a central nexus linking SMN deficiency to NMJ destabilization and neuromuscular degeneration, bridging cell-intrinsic and intercellular pathogenic mechanisms. Key experimental and clinical studies supporting these mechanistic links are summarized in Table 3, integrating molecular alterations, model systems, and functional outcomes across SMA research.

### 4.3. Candidate Molecules and Pathways

Several molecular classes exemplify this model (Table 4). Muscle-specific microRNAs (myomiRs)—miR-206, miR-133b, miR-23a/b—orchestrate NMJ maturation, AChR clustering, and muscle catabolism by modulating the DOK7–CRK–RAC1 signaling cascade, essential for postsynaptic stabilization [113,114]. Disruption of this regulatory axis drives NMJ disorganization and muscle atrophy, consistently observed across SMA models with altered myomiR expression. Notably, miR-206 is consistently upregulated in SMA, ALS, and SBMA as an endogenous compensatory response; yet this induction remains insufficient to restore motor neuron integrity or halt disease progression [115].

Proteins such as Agrin and SNAP-25, normally trafficked within motor neuron–derived exosomes, are equally critical. SMN deficiency reduces Agrin—especially the Z+ isoform—compromising NMJ maturation and stability, whereas its restoration improves NMJ architecture and muscle innervation in preclinical SMA models [109,116].

Finally, trophic factors including BDNF, GDNF, and IGF-1 sustain neuronal and muscular health under physiological conditions [117,118,119]. Their exosomal packaging enables bidirectional signaling between motor neurons, muscle fibers, and glia. Loss of these factors from vesicular cargo—whether via impaired biogenesis, metabolic stress, or neurodegeneration—deprives the NMJ of essential protective inputs, shifting exosomes from stabilizing vectors toward deficient messengers that fail to support neuromuscular resilience [26,120,121].

### 4.4. Translational Implications: Biomarkers and Therapeutics

Positioning exosomal dysfunction at the center of SMA pathophysiology carries immediate translational relevance. Exosomes circulate in CSF and blood, offering a minimally invasive window into disease biology unavailable for nuclear splicing defects confined within cells [26,122,123].

SMN protein itself is packaged into exosomes, with levels reduced in SMA patients versus controls and correlating with genotype and clinical severity [107]. Exosomal microRNA profiles likewise track motor neuron health and respond dynamically to nusinersen therapy, validating their role as biomarkers for treatment response and residual disease activity [54]. Dynamic shifts in exosomal cargo—spanning miRNA signatures, synaptic proteins, and trophic factors—closely track therapeutic efficacy and disease progression, linking molecular changes to clinical outcomes [108,124].

Beyond diagnostics, exosomes constitute therapeutic vectors. Engineered vesicles delivering miR-206, Agrin fragments, or neurotrophic factors such as GDNF and IGF-1 restore lost signaling at the NMJ, stabilizing synaptic architecture and promoting muscle resilience [125,126,127]. Complementary strategies include modulating endogenous vesicle pathways—enhancing release or uptake of beneficial exosomes while inhibiting pathogenic signaling—to rebalance intercellular communication [128].

Pharmacological targeting of Rab GTPases or ESCRT components represents another mechanistically distinct approach. Modulating Rab5, Rab7, or their regulatory proteins (GEFs, GAPs, GDIs) corrects cargo missorting and restores endosomal trafficking fidelity in neurodegeneration models, offering synergy with SMN-restorative therapies rather than competition [10,88,129].

### 4.5. Toward a Dual-Therapy Paradigm

SMN-directed therapies—antisense oligonucleotides, small molecules, gene replacement—correct the upstream genetic defect, prolonging motor neuron survival. Yet residual synaptic and intercellular dysfunction persists when treatment is delayed or incomplete [103,130].

Exosome-targeted approaches address this downstream pathology, repairing molecular communication at the NMJ and within neural networks. Preclinical studies show that exosome-mediated delivery of SMN protein or neurotrophic factors restores nuclear SMN localization and synaptic function, supporting combined therapeutic strategies integrating genetic rescue with restoration of intercellular signaling [81].

Consensus in the literature supports this dual-therapy model, targeting both SMN-dependent and SMN-independent pathways to achieve durable, comprehensive disease control beyond what any single intervention can provide [81].

## 5. Therapeutic Approaches Targeting Axonal Exosomal Signaling

Exosomes, small EVs, carrying proteins, lipids, and nucleic acids, have emerged as highly adaptable delivery systems evolved to mediate intercellular communication efficiently. Their capacity to cross biological barriers, protect labile cargo, and transmit multimodal molecular signals provides a compelling rationale for therapeutic engineering. In SMA, where SMN-deficient motor neurons fail to deliver essential trophic cues, engineered exosomes or modulation of endogenous exosome secretion could restore the disrupted molecular dialogue between axons and muscle fibers [131].

Among exosomal cargo, regulatory microRNAs (miR) are leading candidates. miR-206 has a central role in NMJ repair in SMA and related disorders, its expression rising in muscle, spinal cord, and serum as a conserved denervation response [114,115,132]. It promotes synaptic maintenance and regeneration, and its exogenous delivery (via viral or exosome vectors) reduces motor neuron loss and improves survival through the HDAC4–FGFBP1 axis and NCX2 regulation [113,133,134]. Yet, endogenous upregulation is insufficient, as loss-of-function accelerates NMJ degeneration in ALS and DMD [113], underscoring the need for spatially controlled augmentation rather than passive observation.

While miR-133b co-localizes with miR-206, evidence indicates that miR-206 dominates NMJ repair [135]. Still, exosomal transfer of miR-133b from mesenchymal stem cells promotes axonal regeneration and neuroprotection in spinal cord injury [136]. Denervated muscle-derived exosomes show increased miR-206 but decreased miR-133a/b, suggesting that disease-associated shifts in muscle-specific miRNAs (myomiR) composition could be therapeutically reprogrammed [137].

Protein- and peptide-based cargoes also reinforce NMJ stability. Agrin fragments (NT-1654) activate the agrin/Lrp4/MuSK pathway, reversing synaptic disassembly and promoting re-innervation [138]. On the presynaptic side, SNAP25, central to vesicular docking and neurotransmitter release, remains a rational preservation target for maintaining NMJ function [139]. Neurotrophic factors, such as BDNF, GDNF, and IGF-1, further modulate neuronal survival and muscle trophism. Through TrkB signaling, BDNF enhances synaptic maintenance; GDNF preserves NMJ structure and motor neuron viability, while IGF-1 exerts metabolic and anti-apoptotic protection [116,140,141,142]. Their inclusion in engineered exosomes offers a multidimensional approach to stabilize synaptic architecture and sustain trophic support.

### 5.1. Engineered Exosomes: Technological Foundations and Cargo Loading Strategies

The translation of exosome-based therapies depends not only on defining effective cargo but also on mastering platforms for their precise loading and delivery. Cargo can be incorporated through endogenous engineering or exogenous post-isolation methods, each with specific trade-offs.

In the endogenous approach, donor cells (e.g., mesenchymal stem cells or induced neurons) are engineered to overexpress desired molecules, ensuring natural incorporation during vesicle biogenesis and retaining post-translational fidelity. However, it requires stable genetic modification and faces scalability and safety concerns [143].

By contrast, exogenous methods, such as electroporation or lipid fusion, enable rapid loading into purified EVs without altering donor cells but risk lower efficiency, aggregation, and compromised vesicle integrity, while the incorporation of complex proteins remains challenging [143].

EVs delivery route optimization adds another layer of challenge. Intrathecal injection bypasses the blood-brain barrier but is invasive, intravenous administration broadens biodistribution yet suffers from rapid clearance, while intramuscular delivery offers NMJ-specific reinforcement but limited reach [144].

In early-phase design, endogenous engineering suits complex, combinatorial cargo requiring biological fidelity, whereas exogenous loading provides flexibility for rapid prototyping. Ultimately, the chosen application route and loading strategy must balance efficiency, specificity, and translational feasibility, guiding iterative development of EV-based therapeutics for neuromuscular disorders [144].

### 5.2. Modulating Endogenous Exosome Biogenesis and Secretion

While engineered exosomes provide an external route, restoring the neuron’s intrinsic vesicle machinery represents a more physiological solution. In SMA, SMN deficiency impairs snRNP biogenesis, SNARE assembly, and vesicle trafficking, disturbing exosome formation and NMJ maintenance. Nusinersen restores SMN, normalizes nuclear structures, corrects splicing, and reestablishes physiological vesicle release [96,145]. Endogenous repair thus provides a sustained, cell-autonomous source of therapeutic vesicles, reducing dosing frequency and immune risk [145].

Key molecular regulators of exosome biogenesis offer further therapeutic leverage. Rab27a/b control multivesicular body trafficking through Rab27a mediating fusion and Rab27b positioning. Their effectors, Slp4 and Slac2b, refine secretion as activating Rab27a/b could restore exosome output in SMN-deficient neurons [146,147]. The ESCRT complex (TSG101, Alix, CHMP1A/B, CHMP5, IST1) governs intraluminal vesicle formation and cargo sorting [148,149] and the pharmacological upregulation of ESCRT factors or Rab27a/b may correct secretion defects [150].

Other promising targets include nSMase2, a ceramide-pathway regulator of vesicle budding [130,151], and HDAC inhibitors such as panobinostat, which enhance SMN2 splicing and modify exosomal miRNA cargo, improving neuronal survival and synaptic plasticity [152,153]. Compounds stabilizing actin or RNA-binding proteins could further normalize trafficking and enhance neuronal resilience [130,151].

A major translational challenge is EVs heterogeneity, as subtypes may acquire maladaptive or pro-inflammatory phenotypes in advanced disease [154,155]. Therapeutic benefit requires selective enrichment of protective vesicles while suppressing harmful subtypes through the following:

- Advanced isolation of regenerative/immunomodulatory vesicles,
- Engineering parental cells or environments to bias output,
- Molecular targeting for precision delivery [154,155,156].

Emerging single-vesicle profiling now allows quantitative subpopulation mapping, vital for safety and efficacy validation [157]. Harmonized international standards are essential for analytics, biosafety, and translational reproducibility [158].

Neither SMN-restorative nor exosome-based strategies alone can achieve full recovery. Combining SMN2 splicing correction or gene therapy with engineered exosomes carrying neurotrophic factors could reinforce NMJ stability and motor neuron survival, while nSMase2 activators or cytoskeletal stabilizers may enhance endogenous vesicle release and the evidence that vesicle-delivered SMN integrates into native complexes supports feasibility of these strategies [81].

Ongoing clinical development of exosome therapeutics in oncology and regenerative medicine demonstrates safety and scalability, yet biodistribution, immunogenicity, and cargo stability remain underexplored in neuromuscular contexts. SMA, however, presents an ideal candidate: a well-defined genetic lesion, measurable biomarkers, and clear unmet clinical need. If integrated with SMN-restorative agents, exosome-based approaches could shift SMA treatment from survival prolongation toward genuine neuromuscular restoration.

Targeting axonal exosomal signaling reframes SMA not only as motor neuron degeneration but as failure of intercellular communication. By reactivating this dialogue–through engineered vesicles, pharmacological modulation, or dual-therapy paradigms–future interventions may achieve durable restoration of neuromuscular connectivity. The next step is to conduct systematic experiments to turn this conceptual feasibility into therapies that could alter the natural progression of SMA.

## 6. Preclinical Experimental Design for Exosomal SMA Therapy

Research on therapeutic interventions for SMA has relied on a spectrum of in vitro and in vivo models. A concise summary of the key preclinical and translational elements is presented in Table 5. In vitro models are dominated by human cell systems, especially patient-derived fibroblasts and induced pluripotent stem cell (iPSC)-derived motor neurons. These models capture the fundamental genetic lesion of SMA, such as the deficiency of full-length SMN protein due to aberrant SMN2 splicing [159,160]. They allow detailed analysis of SMN splicing correction, protein restoration, nuclear body formation, axonal growth, and neuromuscular junction maturation. Advances in three-dimensional spinal organoids and motor neuron-myotube co-cultures have further improved validity, enabling the study of motor unit connectivity and synaptic function [161]. Such systems are invaluable for high-throughput testing of antisense oligonucleotides, small molecules, or exosome-delivered cargos. However, they cannot model pharmacokinetics, biodistribution, systemic immune responses, or the complexity of motor unit degeneration across tissues, which necessitates progression to animal studies.

In vivo models provide the necessary systemic context. A key limitation of all animal models is that, unlike humans, most other species lack a native SMN2 gene [162]. This means that disease-relevant splicing regulation has to be artificially introduced by human transgenes, and the expression level and insertion site of these transgenes can vary between colonies, leading to differences in disease severity and treatment responsiveness [163,164]. As a result, while these models reproduce many features of SMA, they do not fully capture the complexity of human SMN2 copy number variation or its impact on disease phenotype and therapeutic outcome. The most widely used severe mouse model, the SMNΔ7 mouse (SMN-/-; SMN2+/+; SMNΔ7+/+), develops rapid-onset motor neuron degeneration, neuromuscular junction pathology, and early death by around postnatal day 14 [163]. This model offers high predictive validity for interventions such as nusinersen-like splice-modulating oligonucleotides and AAV9-SMN1 gene therapy, both of which extend survival in mice and patients alike [165]. Intervention endpoints include survival, body weight, motor behaviors (righting reflex, tail suspension), electrophysiology (compound muscle action potentials, motor unit number estimation), molecular restoration of SMN, and histological analyses of motor neurons, neuromuscular junctions, and muscle fibers. Intermediate models such as Smn2B/-, which harbors one null allele (Smn-) and one engineered murine allele (Smn2B) containing a point mutation in exon 7 that disrupts splicing, extend lifespan into adolescence or adulthood, allowing longitudinal functional assessments such as rotarod performance, grip strength, and progressive neuromuscular junction degeneration [166]. These models are particularly useful for evaluating the durability of effect, the safety of repeat dosing, and the therapeutic benefit after symptom onset, though larger cohorts are required to detect incremental improvements. Large-animal studies, especially in pigs and non-human primates, do not model SMA genetics but are critical for evaluating clinically relevant delivery routes such as intrathecal and intravenous administration, biodistribution within the central nervous system, and potential toxicity to dorsal root ganglia or systemic organs [167].

At present, the potential of exosomes for SMA therapy is primarily limited to preclinical studies. The landmark paper in 2011 showed that dendritic cell-derived exosomes engineered to display the RVG peptide fused to Lamp2b could deliver siRNA across the BBB after systemic administration in mice, resulting in effective knockdown of BACE1 and GAPDH transcripts with minimal immunogenicity [168]. Subsequent work refined this RVG-exosome strategy and confirmed that CNS-targeted exosomes can deliver therapeutic oligonucleotides to neurons after peripheral dosing [169,170]. In neurodegenerative disease models, for example, RVG-exosomes carrying siRNA or shRNA against α-synuclein reduced protein aggregation and pathology in transgenic Parkinson’s disease mice [170], while other studies have reported functional delivery of catalase mRNA to the brain with neuroprotective outcomes [171]. These studies demonstrate that EVs can transport both small and large nucleic acids into the central nervous system. Translating this approach to SMA, exosomes could, in principle, be loaded with SMN1 mRNA to provide transient restoration of protein expression, or with ISS-N1 antisense oligonucleotides to correct SMN2 splicing in motor neurons. Preclinical demonstration of this concept in SMA models has not yet been published, but the strong precedent in other neurodegenerative disease models suggests feasibility. Such engineered vesicles would complement both protein-loaded EVs and unmodified stem cell EVs, broadening the therapeutic landscape for SMA.

SMN protein is naturally secreted in EVs derived from human fibroblasts and detectable in serum EVs, and the levels are lower in SMA cells or patients, an observation that initiated the concept of EVs as SMN biomarkers and as delivery vehicles for replacement therapy [107]. More recently, René and Parks (2025) demonstrated that EVs harvested from HepG2 donor cells overexpressing SMN could transfer functional SMN protein into A549 cells and SMA patient fibroblasts. The imported protein was correctly localized to nuclear bodies and co-localized with Gemin2, suggesting physiological functionality. However, this work remains limited to in vitro studies; no in vivo efficacy, biodistribution, or pharmacokinetic analyses have yet been reported, and no physiological outcomes beyond molecular localization were demonstrated [81].

The most compelling in vivo data for SMA to date involve mesenchymal- or adipose-derived stem cell EVs acting through SMN-independent mechanisms. In SMNΔ7 mice, intracerebroventricular injection of EVs derived from human adipose tissue-derived stem cells (hASCs) improved righting reflexes and other early behavioral measures, increased lumbar motor neuron survival, reduced cleaved caspase-3 expression, and attenuated astroglial activation [172]. These experiments included PBS-treated SMA mice and wild-type littermates as controls, and the EVs were characterized by transmission electron microscopy, nanoparticle tracking analysis, and marker expression (CD9, HSP70). Proteomic analysis indicated enrichment in proteins associated with trophic and anti-apoptotic activity, such as IGF-1 and antioxidant enzymes (SOD1, SOD3), as well as pathway signatures implicating PI3K-Akt signaling. Importantly, no full-length SMN was detected in these EVs, possibly reflecting either low SMN expression in MSCs or exosome-sorting differences [173], suggesting a mechanism based on pleiotropic or immunomodulatory support rather than SMN replacement.

Taken together, current research positions EVs for SMA therapy on two tracks: engineered vesicles carrying SMN1 mRNA, antisense oligonucleotides, or SMN protein itself, and unmodified stem-cell-derived vesicles that exert SMN-independent neurotrophic or anti-inflammatory effects. But both approaches present significant translational hurdles. The majority of efficacy studies so far rely on neonatal intracerebroventricular dosing, a route that is useful for proof-of-concept but less clinically practical compared to intrathecal or systemic delivery in older animals [169,171]. Quantitative exposure of EV cargo in the CNS and at motor units is poorly characterized, and artefactual biodistribution data from lipophilic dyes may complicate interpretation [174]. More rigorous methods, such as genetic labeling of vesicle membranes or cargo, quantitative PCR and ELISA in CNS and peripheral tissues, and sampling of cerebrospinal fluid and plasma, will be needed to build reliable pharmacokinetic-pharmacodynamic relationships [175].

Quality in extracellular vesicle preparations also complicates their application as therapeutic products. Conventional laboratory approaches such as differential ultracentrifugation remain widely used in preclinical studies but are poorly suited to clinical translation: they are labor-intensive, low-throughput, and prone to co-isolation of protein aggregates and lipoproteins [176]. Density-gradient ultracentrifugation improves purity but is even less practical at scale and introduces additional processing reagents that require removal. Polymer precipitation methods provide higher yields but lack specificity, bringing substantial contamination and variable biological activity [177]. For translational applications, more reproducible methods are increasingly preferred. Size-exclusion chromatography (SEC) allows gentle separation of vesicles from soluble proteins and has become a common polishing step [178]. Tangential-flow filtration (TFF) is now widely adopted in clinical-grade workflows because it enables concentration of large culture volumes, efficient buffer exchange, and integration into closed, scalable systems [179]. Chromatographic approaches, including anion-exchange and multimodal resins, can further improve purity by removing nucleic acids, host-cell proteins, and serum-derived contaminants [180]. While immunoaffinity capture using antibodies against exosome markers (e.g., CD63, CD81) yields highly pure populations, it remains expensive and technically difficult to implement at therapeutic scale, and is therefore more useful as an analytical or quality-control tool [181].

Scaling up production requires not only suitable purification but also upstream process design. EVs intended for therapy are typically produced in serum-free or EV-depleted culture conditions, often using large-scale cell expansion systems such as hollow-fiber bioreactors or stirred-tank systems with microcarriers [182,183]. These platforms allow continuous harvest of conditioned medium, which can then be clarified, concentrated by TFF, and polished by SEC or chromatography in a manner compatible with GMP standards. Such closed, single-use systems reduce contamination risk and provide batch-to-batch consistency, both of which are critical for regulatory acceptance.

While a 2024 meta-analysis of human EV trials indicates that EV therapies are generally safe, indication-specific efficacy remains to be proven for CNS diseases such as SMA [184]. Defining release criteria for EV therapeutics is equally important. Beyond particle concentration and size distribution (by nanoparticle tracking or resistive pulse sensing), regulatory-grade assays require marker panels confirming vesicle identity (e.g., CD9, CD63, CD81 positivity; calnexin negativity), endotoxin and sterility testing, residual host-cell protein and DNA quantification, and a functional potency assay relevant to the intended indication. For SMA, such potency assays might include restoration of exon 7 inclusion and gem-body formation in SMA iPSC-derived motor neurons, or survival and neurite outgrowth in motor neuron cultures. For SMN-independent preparations such as mesenchymal stem cell EVs, functional readouts of anti-apoptotic or trophic signaling pathways (e.g., PI3K–Akt activation) would be more appropriate.

## 7. Clinical and Translational Implications

The introduction of exosome-based therapeutics into the treatment landscape of SMA is a promising avenue for addressing some limitations of current therapy. Even though the treatment of SMA has been dramatically improved through gene replacement, ASO, and small-molecule splicing, these strategies do not provide a complete cure. Several challenges remain: heterogeneous patient responses, variable penetrance of therapy into motor neurons, partial restoration of neuromuscular function, and continued disease progression in many individuals despite treatment [185,186,187,188,189]. It is clear that we must look beyond SMN correction for possible adjunctive therapies. The introduction of exosome-based therapeutics into the treatment landscape of SMA is a promising avenue for addressing limitations of current SMN-targeted therapies. Importantly, many of the translational considerations discussed here build upon the preclinical foundation summarized in Table 5.

A recent study by René and Parks [81] demonstrated that exosomes generated from HepG2 cells overexpressing SMN can be loaded with SMN protein, released, and then taken up by recipient cells. The exogenous SMN localized to the nucleus and co-localized with Gemin2, forming nuclear “gem-like” structures, suggesting at least partial functional integration. Therefore, there is a definite translational potential of delivering SMN via naturally derived vesicles rather than, or in addition to, genetic or small molecule tools.

Recent reviews show that vesicle-based therapies are moving beyond speculation and into clinical testing. One review highlights progress, challenges, and standardized protocols in bringing treatments closer to trials, especially in tissue repair, immune regulation, and neuroprotection [190]. Furthermore, there is a clear role for exosomes as delivery vehicles, modulators of inflammation, and carriers of non-coding RNAs, with possible pitfalls like heterogeneity, production at scale, and unintended effects [191]. Still, the fact that such approaches are already being tested in experimental models of stroke, multiple sclerosis, and Alzheimer’s disease makes the connection to spinal muscular atrophy particularly compelling. Neurodegenerative diseases share mechanisms of neuronal vulnerability, synaptic dysfunction, and chronic inflammation, regardless of the initial cause for the damage. Therefore, insights from other neurodegenerative diseases can directly inform the development of vesicle-based strategies aimed at protecting motoneurons and preserving the neuromuscular junction (NMJ) in spinal muscular atrophy.

From a clinical perspective, exosome-based approaches could be envisioned in several scenarios. First, as adjunctive therapy, engineered exosomes carrying trophic factors, miRNAs, or protective proteins could be combined with SMN-restorative treatments to synergistically enhance outcomes. This dual approach would address both the genetic root cause and the secondary pathophysiological consequences of SMA, such as NMJ instability and progressive denervation [54,81,190,192]. Second, as a standalone therapy, exosome-based treatment could serve patients who fail to respond adequately to SMN-targeted drugs, or those in advanced disease stages where neuronal loss has already occurred and muscle integrity becomes a primary therapeutic target. Finally, in the preventive setting, exosomes could be administered in presymptomatic infants identified through newborn screening programs, with the goal of reinforcing NMJ maturation and delaying symptom onset [172].

Translation of such strategies into clinical practice requires careful navigation of regulatory and manufacturing considerations. Exosomes intended for therapeutic use must be produced under good manufacturing practice (GMP) conditions, with defined standards for purity, reproducibility, and stability [158,193]. Equally important is the development of assays for quality control, including particle characterization, cargo profiling, and potency testing. These challenges parallel those encountered during the development of cell and gene therapies, and regulatory authorities such as the FDA and EMA are already laying the groundwork for evaluating extracellular vesicle-based interventions [158].

Designing early-phase clinical trials will be essential to establish safety, tolerability, and proof-of-concept efficacy. Phase I studies would likely focus on dose-escalation and biodistribution in small patient cohorts, employing outcome measures such as pharmacokinetics of exosome cargo, serum biomarkers (e.g., neurofilament light chain, disease-relevant miRNAs), and safety endpoints [193]. Recent study by Virla et al. highlights the promising results of using exosomes using an in-vivo mouse model [172]. Subsequent Phase II/III trials could expand to efficacy evaluations, incorporating functional motor scales (CHOP-INTEND, HFMSE), electrophysiological measures (CMAP amplitudes), and imaging modalities (muscle MRI) as objective markers of clinical benefit [194]. Finally, patient stratification is also a key factor; presymptomatic infants, early-onset SMA type I patients, and later-onset types II and III may all require distinct trial designs and therapeutic goals [195].

Beyond the scientific and regulatory aspects, ethical and economic considerations will play a decisive role in clinical translation. SMA therapies are among the most expensive in modern medicine, with single-dose gene therapy costing millions of dollars per patient [17,195]. If scalable exosome production methods can be developed, there is potential for substantially reducing costs relative to current standards, thereby expanding global access to innovative care [191]. Ethically, clinicians and regulators will need to consider the long-term implications of manipulating endogenous nanocarriers, particularly regarding unforeseen effects on intercellular communication in non-target tissues. Transparent risk–benefit assessment and patient-centered trial design will be essential for ensuring societal trust and acceptance [196].

Finally, exosome profiling itself may emerge as a biomarker platform to guide clinical decision-making. By analyzing circulating exosomes from SMA patients, clinicians could identify signatures of disease progression or treatment response, enabling a more personalized therapeutic approach [107]. The ability to track whether circulating EVs in treated patients carry functional SMN, as suggested by René et al., could become an especially valuable biomarker for therapeutic effectiveness [81].

In summary, the integration of exosome-based therapies into SMA management holds transformative potential. Their unique biological properties offer an opportunity to complement existing SMN-targeted treatments, address unmet clinical needs, and pave the way toward more personalized and durable outcomes. The path to translation will require coordinated efforts spanning bench-to-bedside research, regulatory harmonization, and ethical reflection, but the reward may be a new therapeutic frontier capable of reshaping the clinical trajectory of SMA.

## 8. Challenges, Open Questions and Future Perspectives

Exosome-based therapeutics for SMA present a compelling but technically demanding frontier. Their major clinical appeal lies in the ability to complement SMN-restorative drugs by directly targeting neuromuscular junction pathology and skeletal muscle integrity, components that remain insufficiently corrected by existing therapies. Given that SMA care already relies on multimodal approaches, an adjunct exosome-based intervention could conceptually integrate well into current management pathways, though rigorous safety and efficacy data will be indispensable [197].

Despite strong preclinical signals in neurodegenerative disorders such as Alzheimer’s and Parkinson’s disease [169,171], exosome efficacy in SMA remains unproven. While engineered vesicles can deliver siRNAs, shRNAs, and mRNAs across the blood-brain barrier with measurable neuronal activity, no comparable in vivo therapeutic demonstration exists for SMA [169,170]. Establishing clear functional benefit in SMA models is therefore essential before clinical translation can proceed.

Major technical hurdles persist. Isolation platforms (ultracentrifugation, polymer precipitation, size-exclusion chromatography, tangential flow filtration) yield vesicle populations with differing purity and cargo preservation [198], influencing biological activity [199] and complicating reproducibility. Upstream variability—including donor heterogeneity, passage number, and differentiation state—further contributes to batch-to-batch differences, while most preparations contain mixed EV subtypes, obscuring the identity of the active fraction.

Unlike single-dose gene therapies, exosome treatment will likely require repeated administration. Determining the optimal dosing interval, delivery route, and long-term safety profile remains critical, especially since most SMA studies rely on neonatal intracerebroventricular injection. Clinically feasible routes (systemic, intrathecal, intramuscular) remain insufficiently characterized. Evidence that intranasally delivered MSC-derived EVs can reach the CNS [200] is encouraging, but robust pharmacokinetic analysis is lacking, and common tracking techniques introduce artefacts. New imaging and tracing platforms—radiotracer-labelled EVs (PET/CT) [201], DNA barcoding [202], genetic reporters [203,204], multimodal PalmGRET [205], and USPIO-enhanced MRI [206]—offer tools capable of establishing reliable in vivo PK/PD relationships.

Biomarker development is equally important. Circulating exosomal signatures, including SMN-containing vesicles and miRNA panels, could enable treatment stratification and serve as surrogate endpoints, yet remain unvalidated in SMA cohorts [190,191]. Cost and accessibility pose additional challenges: current SMA therapies already impose a substantial economic burden, underscoring the need for scalable, GMP-compliant manufacturing pipelines for exosome therapeutics [17]. Ethical considerations—particularly transparent communication with families regarding off-target biodistribution and vesicle-pathway manipulation—must be integrated into early clinical planning.

Mechanistic uncertainty remains a central translational barrier. Benefits observed with MSC-EVs in SMA mice are frequently described as ‘SMN-independent’ [172] because earlier proteomic analyses did not detect SMN cargo [207]. However, shotgun proteomics lacks sensitivity to low-abundance proteins, SMN mRNA was not assessed, and functional delivery to motor neurons has never been directly quantified. Whether MSC-EVs exert true SMN-independent rescue or provide subtle SMN-restorative activity alongside broader trophic and immunomodulatory effects therefore remains unresolved.

Progress will depend on bridging mechanistic and clinical insight. Human iPSC-derived neuromuscular organoids now permit high-fidelity modelling of motor neuron-muscle interactions [208,209]. Grass et al. [210] demonstrated that isogenic SMA and CRISPR-corrected organoids recapitulate disease-relevant neurodevelopmental abnormalities, providing platforms capable of dissecting exosome mechanisms and optimizing therapeutic design.

In summary, exosomes represent a promising therapeutic axis for SMA, but their adoption will require resolving key preclinical uncertainties, demonstrating additive benefit over current SMN-restorative interventions, establishing pediatric safety, and ensuring scalable and equitable access. Researchers must define mechanism and potency; clinicians will need to integrate these therapies into holistic, individualized care without increasing the burden on families or healthcare systems.

## 9. Conclusions

Exosome-based modulation of axon-to-muscle communication offers a promising adjunct to current SMN-restorative therapies in SMA. Available preclinical evidence shows that vesicle-mediated delivery of SMN protein, neurotrophic factors, and regulatory microRNAs can stabilize neuromuscular junctions, reduce inflammation, and support motor neuron survival. However, successful translation will require standardized vesicle isolation, rigorous characterization, and optimized dosing strategies, together with robust safety evaluation. Integrating exosome-targeted approaches with gene replacement or splicing correction may ultimately provide a more comprehensive therapeutic effect than either modality alone. Future studies employing human iPSC-derived neuromuscular organoids, GMP-compatible EV production, and biomarker-driven clinical trial designs are essential to determine clinical feasibility and therapeutic value.

## Figures and Tables

**Table 1:** Natural history-based clinical classification of spinal muscular atrophy.

SMA Type	Age at Onset	Maximum Motor Function	Typical Prognosis
**0**	Prenatal	No motor function	Severe hypotonia at birth; death usually <6 months
**1**	<6 months	Never sits	Life expectancy rarely exceeds 2 years
**2**	7–18 months	Sits unsupported	Lives into adolescence or early adulthood
**3**	>18 months	Walks independently	Slow progression; normal or near-normal lifespan

Note: Before the availability of disease-modifying therapies, SMA was categorized into five types (0–4) based on age at symptom onset, highest motor milestone achieved, and typical prognosis.

**Table 3:** Key Experimental and Clinical Evidence Linking Exosomal Dysfunction to SMA Pathophysiology.

Molecular/Pathway Focus	Study (Year)	Model/Population	Key Findings	Relevance to Hypothesis
**SMN protein in exosomes**	Nash et al., 2017 [107]	SMA serum/CSF	SMN packaged into circulating exosomes; reduced in SMA; correlates with genotype and severity.	EV cargo mirrors disease burden; SMN-in-exosomes is a biomarker of residual disease activity.
**miR-206, miR-133a-3p response to therapy**	Magen et al., 2022 [108]	SMA II/III on nusinersen (longitudinal CSF)	miR-206/133a-3p inversely correlate with motor score; early predictors of therapeutic response.	EV/CSF miRNAs act as dynamic biomarkers of NMJ health and drug efficacy.
**Agrin Z+ isoform restoration**	Kim et al., 2017 [109]	SMA mouse models	SMN deficiency lowers neuronal Z+ Agrin; restoration enlarges NMJ, improves innervation, increases fiber size, extends survival.	Loss of neuronal synaptic cues destabilizes NMJs; restoring cargo is disease-modifying.
**Agrin mimetic NT-1654**	Boido et al., 2018 [110]	SMA mice	NT-1654 enhances NMJ maturation, reduces denervation, improves outcomes.	Proof that replacing missing synaptic organizers rescues NMJ architecture.
**Presynaptic vesicle machinery**	Tejero et al., 2016 [111]	SMA (SMNΔ7) mice	Syt2, SV2B markedly reduced in vulnerable muscles; impaired neurotransmitter release.	Presynaptic protein loss drives NMJ instability; aligns with cargo-deficiency hypothesis.
**Axonal trafficking nodes (Rab5/Rab7)**	Deinhardt et al., 2006 [112]	Motor neurons, DRG	Rab5 regulates early endosomes; Rab7 long-range retrograde transport; Rab7 inhibition blocks cargo transport.	SMN-linked trafficking deficits may impair Rab5→Rab7 transition, altering exosomal sorting.
**Exosomal SMN delivery**	René et al., 2025 [81]	SMA in vitro models	EV-delivered SMN restores nuclear SMN localization; functional rescue.	Confirms EVs as therapeutic vectors supporting the axon-to-muscle repair hypothesis.

SMN—survival motor neuron; SMA—spinal muscular atrophy; CSF—cerebrospinal fluid; EV—extracellular vesicle; NMJ—neuromuscular junction; miR—microRNA; Syt1/Syt2—synaptotagmin-1/-2; SV2B—synaptic vesicle glycoprotein 2B; DRG—dorsal root ganglion.

**Table 4:** Key molecular candidates in exosomal signaling as biomarkers and therapeutic targets for SMA.

Molecule	Normal Function in NMJ/Muscle	Predicted Alteration in SMA Exosomes	Expected Consequence
**miR-206**	Promotes NMJ regeneration, supports reinnervation after injury	Reduced neuronal exosomal delivery (compensatory muscle upregulation insufficient)	Delayed, incomplete NMJ repair; partial synaptic stabilization
**miR-133b**	Regulates acetylcholine receptor clustering and postsynaptic differentiation	Loss from motor neuron exosomes	Fragmented and unstable postsynaptic architecture
**miR-23a/b**	Protects against muscle atrophy via inhibition of catabolic pathways	Underrepresented in SMN-deficient exosomes	Accelerated muscle wasting
**Agrin/MuSK modulators**	Induce acetylcholine receptor clustering and NMJ maturation	Deficient exosomal transfer	Impaired synaptic assembly, immature NMJs
**Synaptophysin, SNAP25**	Support vesicle docking and neurotransmitter release	Reduced delivery to synaptic sites	Presynaptic disassembly, impaired neurotransmission
**BDNF**	Enhances motor neuron survival and synaptic stability	Lower exosomal release	Reduced trophic support, synaptic vulnerability
**GDNF**	Strengthens NMJ connectivity and motor neuron resilience	Loss from exosomal cargo	Weakening of neuromuscular signaling, denervation
**IGF-1**	Promotes muscle growth, regeneration, and metabolic resilience	Diminished exosomal presence	Reduced muscle repair capacity, enhanced atrophy

NMJ—neuromuscular junction; miR—microRNA; SMN—survival motor neuron; MuSK—muscle-specific kinase; SNAP25—synaptosomal-associated protein 25; BDNF—brain-derived neurotrophic factor; GDNF—glial cell line-derived neurotrophic factor; IGF-1—insulin-like growth factor 1.

**Table 5:** Summary of Key Preclinical and Translational Elements for Exosome-Based SMA Therapy.

Domain	Key Models/Approaches	Main Findings	Translational Relevance
**In vitro SMA systems**	Patient fibroblasts; iPSC-derived motor neurons; organoids	SMN restoration, improved axonal growth and NMJ maturation	Fast screening of exosome cargo and potency assays
**In vivo SMA models**	SMNΔ7 mice; Smn2B/-mice	Survival extension, improved motor outcomes with EV-based interventions	Predictive for early-dose strategies and repeat dosing evaluation
**Engineered exosomes**	RVG-targeted EVs; OLIGO- or mRNA-loaded EVs	CNS delivery after peripheral dosing; gene silencing; neuroprotection in other neurodegenerative models	Feasibility of SMN1 mRNA or ASO-loaded exosomes
**Endogenous EV-SMN** **biology**	Human fibroblast EVs; serum EVs from SMA patients	SMN detectable and reduced in SMA; correlates with severity	Biomarker potential and rationale for EV-SMN replacement
**Stem cell EV therapies**	MSC/hASC EVs in SMA mice	Motor improvement, reduced apoptosis and gliosis	SMN-independent therapeutic pathways (trophic and anti-inflammatory)
**Delivery considerations**	Intranasal, intrathecal, intravenous	Effective CNS penetration demonstrated in ALS/PD models	Guides route selection for SMA clinical trials
**Manufacturing and Quality** **Control**	SEC, TFF, bioreactors	Higher purity, scalable production	Required for GMP-grade EV therapeutics
**Clinical** **application**	Adjunctive or standalone therapy; biomarker monitoring	Potential to enhance SMN-targeted drugs and aid non-responders	Supports development of future early-phase trials

## Data Availability

No new data were created or analyzed in this study.
